# The new frontier: a case for whole exome sequencing with multiple fetal anomalies

**DOI:** 10.1515/crpm-2022-0032

**Published:** 2023-05-04

**Authors:** Jenny Y. Mei, Lila Dayani, Lawrence D. Platt

**Affiliations:** Division of Maternal-Fetal Medicine, Department of Obstetrics and Gynecology, University of California, Los Angeles, CA, USA; Laboratory Corporation of America, Monrovia, CA, USA; Center for Fetal Medicine and Women’s Ultrasound, Los Angeles, CA, USA

**Keywords:** fetal anomalies, genetic counseling, ultrasound, whole exome sequencing

## Abstract

**Objectives:**

Standard genetic testing can fail to identify an underlying genetic etiology in pregnancies affected by multiple fetal abnormalities. Recently, whole exome sequencing (WES) studies have shown promise in recognizing genetic diagnoses where standard genetic testing does not yield answers.

**Case presentation:**

A 35-year-old G1P0 healthy female found at anatomy scan to have multiple fetal anomalies, including severe bilateral ventriculomegaly, renal pyelectasis, and short long bones. Karyotype and microarray were normal. Whole exome sequencing showed the fetus was compound heterozygous for likely pathogenic variants in the ROBO1 gene.

**Conclusions:**

In the presence of multiple fetal anomalies with normal karyotype and microarray, whole exome sequencing should be considered to not only provide answers for the affected parents, but also aid in future pregnancy planning.

## Introduction

Fetal abnormalities are identified in 2–5 % of pregnancies, and pregnancy outcome and prognosis can vary greatly depending to the types of abnormalities detected, whether they are isolated or multiple, and if there is an underlying genetic etiology [1]. Using established cytogenetic and molecular techniques, prenatal genetic diagnosis identifies an underlying genetic etiology in around 40 % of fetuses with structural anomalies, leaving the majority of cases undiagnosed [2, 3].

Several prenatal whole exome sequencing (WES) studies, including a recent systematic review and meta-analysis, have identified genetic diagnoses in fetuses with structural abnormalities when standard genetic testing was not diagnostic [4, 5]. In these cases, WES can add clinically relevant information that can assist not only current management of a pregnancy, but also future pregnancy planning. We present a case of a patient with multiple fetal anomalies who had normal karyotype and microarray, and the decision to pursue WES not only yielded important information for the future, but also provided closure for the current pregnancy.

## Case presentation

A 35-year-old Caucasian female G1P0 presented for routine obstetric care. Her spontaneously conceived pregnancy was complicated by advanced maternal age, for which she had a low risk noninvasive prenatal screening and normal Smith-Lemli-Opitz and neural tube defect screening. Her expanded carrier screening showed she was a carrier for factor XI deficiency, otherwise negative for 421 diseases. The father of the pregnancy was 33-years-old and in good health; he was not a carrier for factor XI deficiency. The patient’s father and sister were affected with factor XI deficiency, but otherwise she had an unremarkable family history. She had a normal first trimester ultrasound with nuchal translucency measurement of 2.68 mm.

**Figure 1:**
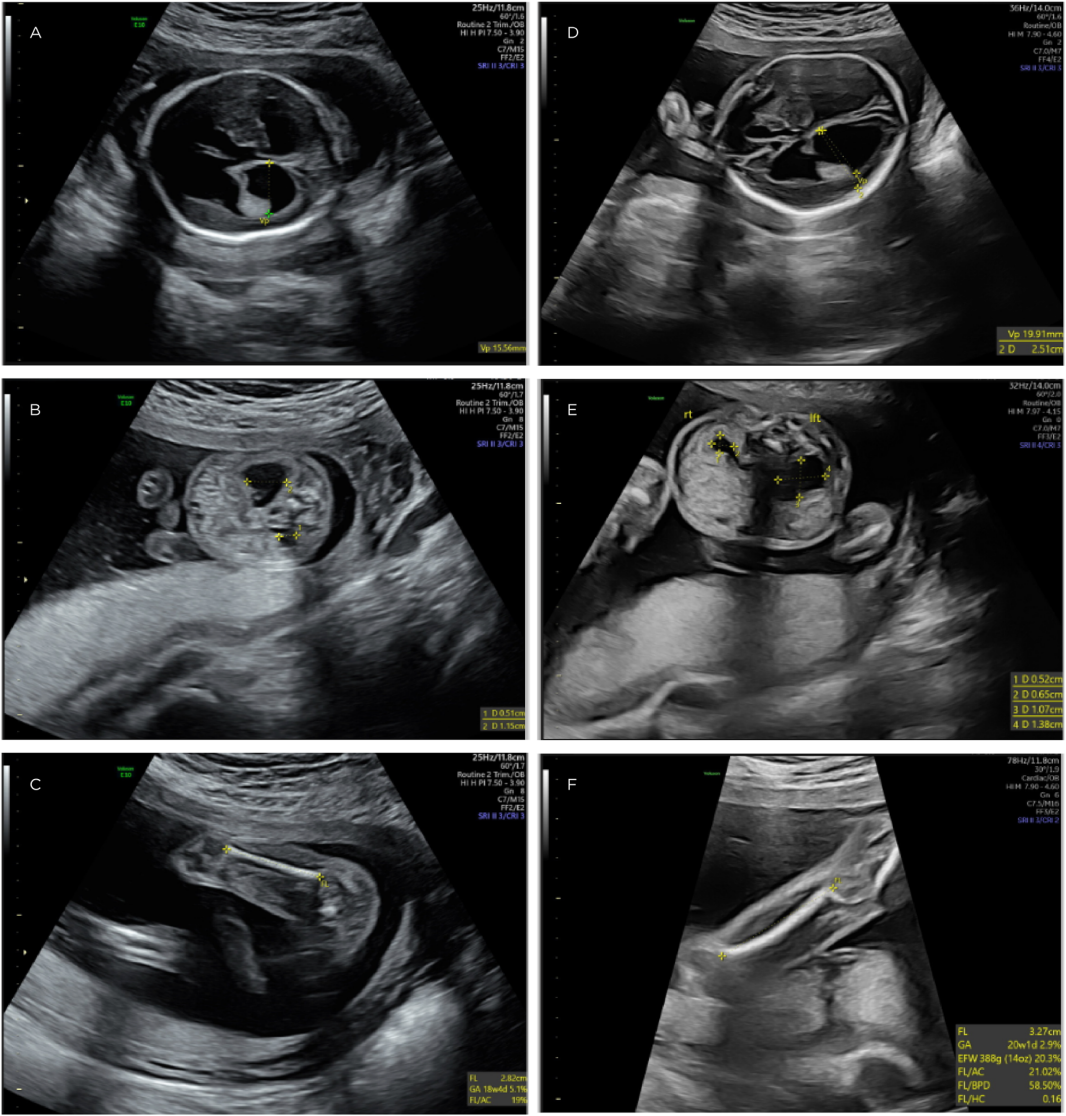
20-week anatomy ultrasound findings compared to 22-week follow-up ultrasound. Transabdominal ultrasound images showing severe bilateral ventriculomegaly (A), renal pyelectasis (B), and short femur (C) length at time of 20-week anatomy ultrasound (left) and worsening in severity at a follow-up 22-week ultrasound (D–F, respectively) (right).

At her anatomy ultrasound at 20w1d, there was notable bilateral ventriculomegaly measuring up to 15 mm, dangling choroid plexus, dilated third ventricle, heart with left axis deviation, and femur length measuring in the 5th% percentile (Figure 1). There was also marked left renal pyelectasis measuring 1.1 cm with a dilated left ureter and hydronephrosis. Following genetic counseling, an amniocentesis was performed which showed 46, XY karyotype and normal microarray. Cytomegalovirus, toxoplasma, and parvovirus IgM and IgG titers were all negative. Of note, the patient did have L1CAM testing included in her carrier screening which came back negative.

The patient subsequently had a fetal brain MRI which corroborated these findings, showing marked dilatation of the lateral ventricles measuring 18–20 mm in diameter. A follow-up ultrasound at 22w0d showed worsening bilateral ventriculomegaly measuring 20 mm, femur length in the 2 % percentile, and worsening left renal pyelectasis with suspected ureteropelvic junction obstruction (Figure 1).

Whole exome sequencing was performed, and the trio analysis (proband and parents) showed the fetus was compound heterozygous for a likely pathogenic variant and a variant of unknown significance (VUS) in the ROBO1 gene (Table 1). The paternally-inherited variant of ROBO1, c.583C>T, is predicted to result in premature stop codon of arginine. This is a nonsense variant and predicted to be pathogenic. The maternally-inherited variant, c.1021G>C, classified as a VUS and results in amino acid substitution from alanine to proline. However, there is an absence of conclusive functional and genetic evidence. Both variants have not previously been reported in literature.

**Table 1:** Whole exome sequencing trio analysis (proband and parents).

Transcript	Mode of inheritance, gene OMIM	DNA variations, predicted effects, zygosity	ClinVar ID	Highest allele frequency in a gnomAD population	In silico missense predictions	Interpretation
*ROBO1,* NM_002941.3	AR, AD, 602,430	c.583C>T, p.Arg195, heterozygous **(paternal)**	Not listed in ClinVar	0.0009 %, European (Non-finnish)	Not applicable	Likely pathogenic
		c.1021G>C, p.Ala341Pro, heterozygous **(maternal)**	Not listed in ClinVar	Not present	Conflicting	Uncertain

Mode of inheritance: AD, autosomal dominant; AR, autosomal recessive; XL, X-linked; ClinVar ID: Variant accession (www.ncbi.nlm.nih.gov/clinvar). GnomAD: Allele frequency registered in a large population database (gnomad.broadinstitute.org). Value listed is the highest allele frequency reported within one of seven population categories recognized in gnomAD v.2.0 (The “Other” population is excluded). Missense predictions: summarized output (damaging, conflicting, or tolerated) via PolyPhen-2, SIFT, MutationTaster, and FATHMM (PMID: 26555599).

The parents opted to terminate the pregnancy. Surgical pathology confirmed the head and kidney findings seen on ultrasound.

## Discussion

ROBO1 (roundabout guidance receptor 1) is a protein coding gene that encodes an integral membrane protein that functions in axon guidance and neuronal precursor cell migration. The receptor itself is activated by SLIT-family proteins that have a repulsive effect on glioma cell guidance in the developing brain [6, 7].

Clinically, variants in ROBO1 have been reported in individuals with developmental disorder, language impairment, epilepsy, and autism [8], [9], [10]. ROBO1 variants have also been reported in association with congenital anomalies of the kidney and urinary tract (CAKUT) [11]. In one family, there were reported compound heterozygous variants in ROBO1 in two fetuses also affected by CAKUT, with both fetuses found to have kidney agenesis, genital hypoplasia, hypoplasia of the halluces, intestinal malrotation, and anteriorly displaced anuses [12].

In our case report, each parent carried a recessive allele of the ROBO1 variant. Given the previously described integral role of ROBO1 in neuronal migration of the developing brain, it is plausible the combination of the two variants were associated with the structural CNS anomalies seen on ultrasound. It is likely the fetus would have been at significant risk for cognitive defects as previously described in literature [8], [9], [10]. It is also possible the ROBO1 variants contributed to the renal anomalies.

The parents were counseled that given the mutations appear to be functioning as recessive alleles, the recurrence risk for future offspring is 25 %. Future pregnancy planning options discussed include prenatal testing via chorionic villus sampling or amniocentesis, or *in vitro* fertilization with preimplantation genetic testing (PGT) for monogenic/single gene conditions to select against ROBO1. They were also counseled that other family members could be carriers of the ROBO1 variants and should be informed of this information and that genetic screening is available.

When karyotype testing and chromosomal microarray fail to determine the underlying cause of a structural anomaly, whole exome sequencing can add clinically relevant information that not only can impact management of the current pregnancy, but also affect future pregnancies. Of note, before jumping to WES, there are specific target gene panels that can identify sets of monogenic disorders if a specific phenotype is known. If there are multiple anomalies or incomplete phenotypic ascertainment, however, the prenatal utility of such panels can be limited, and WES should be considered [13].

Next generation WES provides much greater resolution than standard genetic testing techniques, down to the single base-pair level. WES has been studied in the clinical setting only in the past few years, but studies so far have already demonstrated a diagnostic yield of 25–30 % in post-natal testing in cases undiagnosed with established methods [5, 14].

More recent studies have suggested that diagnostic yields for prenatal WES are higher in fetuses with multiple anomalies or in cases preselected following genetic review [15, 16]. In a prospective cohort study by Petrovski et al. using prenatal WES trio sequence data at a large academic center, diagnostic genetic variants were identified in 10 % of cases affected by a structural anomaly on prenatal ultrasound [17]. The PAGE cohort study by Lord et al. found that the overall detection rate of diagnostic genetic variants in a prospectively ascertained cohort was lower than previously suggested and recommended careful case selection to maximize clinical utility [18].

Accurate fetal phenotyping is key in ensuring highest yield for WES. He et al. found that WES especially added value in genetic diagnosis for abnormal fetuses with skeletal diseases with normal karyotype and microarray [19]. In another study looking specifically at Jewish families, WES yield was relatively higher (42.9–60 %) among families with involvement of brain, renal or musculoskeletal ultrasound findings, especially with a positive family history [20]. A recent systematic review and meta-analysis by Mellis et al. showed diagnostic yield was significantly higher for cases pre-selected for likelihood of monogenic etiology and ranged significantly between phenotypic sub-groups, with highest for isolated skeletal abnormalities at 53 % [5]. Multidisciplinary review may be useful in determining if a monogenic cause is likely and optimizing the expected diagnostic yield of cases.

It is important to note that while WES can improve detection of clinically important variants, it will also increase detection of incidental findings and variants of uncertain significance which can be challenging to interpret. In one study, up to 15 % of abnormal fetuses with normal karyotype and microarray who underwent a WES were found to have VUS [19].

WES also has a lengthy turnaround time, high cost, and requires analysis by a multidisciplinary team of perinatal practitioners, geneticists, and laboratory specialists which presents feasibility challenges. Insurance coverage is challenging especially if the pregnancy has been terminated. Prohibitive costs and challenges with insurance can cause resistance to obtaining WES, which impedes its utilization. In our case, coverage was only approved after extensive peer-to-peer review, and ultimately the findings helped the family immensely for future pregnancy planning. We hope that as the technology improves and the clinical importance of WES becomes more widely recognized, it will be more universally offered and utilized.

Given the high potential for clinical utility in the presence of multiple fetal anomalies with normal karyotype and microarray, whole exome sequencing should be strongly considered in these cases. The whole exome sequencing trio analysis in this case afforded the results needed to bring closure for the parents and aid in future pregnancy planning.
